# Addressing the Joint Impact of Temperature and pH on *Vibrio harveyi* Adaptation in the Time of Climate Change

**DOI:** 10.3390/microorganisms11041075

**Published:** 2023-04-20

**Authors:** Kaan Gundogdu, Ander Orus Iturriza, Maite Orruño, Itxaso Montánchez, Harkaitz Eguiraun, Iciar Martinez, Inés Arana, Vladimir R. Kaberdin

**Affiliations:** 1 Department of Immunology, Microbiology and Parasitology, University of the Basque Country UPV/EHU, 48940 Leioa, Spain; kaan.gundogdu@windowslive.com (K.G.); anderorus15@gmail.com (A.O.I.); maite.orruno@ehu.es (M.O.); itxaso.montanchez@ehu.eus (I.M.); ines.arana@ehu.eus (I.A.); 2 Research Centre for Experimental Marine Biology and Biotechnology (PIE-UPV/EHU), 48620 Plentzia, Spain; harkaitz.eguiraun@ehu.eus (H.E.); iciar.martinez@ehu.eus (I.M.); 3 Department of Graphic Design & Engineering Projects, Faculty of Engineering in Bilbao, University of the Basque Country UPV/EHU, 48013 Bilbao, Spain; 4 IKERBASQUE, Basque Foundation for Science, Maria Diaz de Haro 3, 48013 Bilbao, Spain; 5 Department of Zoology and Animal Cell Biology, Faculty of Science and Technology, University of the Basque Country UPV/EHU, 48940 Leioa, Spain

**Keywords:** climate change, ocean acidification, *Vibrio* survival, coccoid-like cells, adaptation strategies

## Abstract

Global warming and acidification of the global ocean are two important manifestations of the ongoing climate change. To characterize their joint impact on *Vibrio* adaptation and fitness, we analyzed the temperature-dependent adaptation of *Vibrio harveyi* at different pHs (7.0, 7.5, 8.0, 8.3 and 8.5) that mimic the pH of the world ocean in the past, present and future. Comparison of *V. harveyi* growth at 20, 25 and 30 °C show that higher temperature per se facilitates the logarithmic growth of *V. harveyi* in nutrient-rich environments in a pH-dependent manner. Further survival tests carried out in artificial seawater for 35 days revealed that cell culturability declined significantly upon incubation at 25 °C and 30 °C but not at 20 °C. Moreover, although acidification displayed a negative impact on cell culturability at 25 °C, it appeared to play a minor role at 30 °C, suggesting that elevated temperature, rather than pH, was the key player in the observed reduction of cell culturability. In addition, analyses of the stressed cell morphology and size distribution by epifluorescent microscopy indicates that *V. harveyi* likely exploits different adaptation strategies (e.g., acquisition of coccoid-like morphology) whose roles might differ depending on the temperature–pH combination.

## 1. Introduction

Oceans play an important role on our planet as they represent almost 90% of the Earth’s ecosystems and are inhabited by a great number of organisms, including a myriad of different prokaryotic species [1]. However, this profound biodiversity has been altered and noticeably reduced due to the ongoing climate change caused by human activities. Climate change is defined as a long-term alteration in the average weather patterns that impact regional and global climates [2]. This change has been mainly caused by global warming, which is manifested by the long-term warming of the Earth’s climate that started in the preindustrial era (between 1850–1900) and is mainly due to fossil fuel burning which increases greenhouse gas emissions, consequently trapping heat in the atmosphere [2]. As most of the excessive heat produced due to human activities has been absorbed by the ocean, the temperature of the sea surface has been rising continuously [3]. As a result, sea surface temperature (SST) has been increasing by 0.078 °C each decade since 1880. The increase has become greater recently, as the current rate of warming has doubled since 1981 [4].

Another negative consequence of climate change consists in an increase in the concentration of atmospheric CO_2_ from 280 parts per million (ppm) to 412.5 ppm since the pre-industrial era [4]. Almost 30% of it has been captured by the global ocean [3], thereby disturbing the equilibrium in the ocean carbonate system [5] and subsequently reducing its average pH from 8.3 to 8.1 [4]. The lower pH can negatively affect the life of marine organisms, including coral reefs, potentially reducing their protection of other species [6,7,8]. Other adverse effects of ocean acidification include inhibition of shell formation of oyster larvae [9], significantly altering the metabolism and physiology of abalone species [10] as well as suppression of invertebrate immunosystem [11,12]. The alterations in fitness and weakening of immune defense can facilitate infection of industrially important organisms, thus not only causing an economic loss in aquaculture [13] but also increasing the risk of disease transmission by the infected animals to consumers [14,15].

In addition to affecting many eukaryotic organisms, climate change also has a profound effect on marine microbiota, including a large number of *Vibrio* spp. which belong to the Vibrionaceae family and are known for their essential role in carbon cycling and host–pathogen interactions. The ability of *Vibrio* spp. to provide characteristic responses to environmental stress has made them attractive model organisms to study the impact of climate change. Previous work revealed that the seasonal variations in environmental parameters, such as temperature and salinity, can greatly affect *Vibrio* abundance and survival strategies [16,17]. Moreover, recent studies of microbial populations in the North Atlantic and the North Sea disclosed a considerable increase in the abundance of *Vibrio* spp. as a consequence of global warming [18,19]. These findings as well as reports documenting the appearance of the new invasive *Vibrio* spp. in different parts of Europe [20] suggest the high adaptability of the *Vibrio* genus to different environments.

To survive under adverse conditions, bacterial cells can employ different survival strategies (for a review, see [21]). For instance, survival under starvation can lead to a reduction of cell size. This morphological change and global reprogramming of gene expression [22] lead to the acquisition of coccoid-like morphology [23] conferring a higher resistance to stress. Moreover, prolonged exposure to various stress factors occasionally promotes the entry of *Vibrio* cells into a state of dormancy defined as a viable but non-culturable (VBNC) state. On one hand, many bacteria that enter this physiological state are unable to exert growth under standard laboratory conditions for a long time. On the other hand, the cells retain some physiological activities and cell integrity [21,24], and, therefore, are considered to be alive [21,24]. Indeed, when the stress is relieved, the VBNC cells can resume normal physiological activities and growth [25].

Although previous studies demonstrated that pH can potentially play a significant role in determining the diversity and interactions of marine microorganisms, there is still little known about the impact of pH on free-living marine vibrios, especially in the context of climate change. Here, we used *V. harveyi,* a well-characterized marine bacterium known for its extensive use as a model organism to study *Vibrio* ecology [17], to study its physiological responses and fitness at different pH that resembles the pH of seawater in the past, present and near future.

## 2. Materials and Methods

### 2.1. Bacterial Strain and Media

All experiments were done with *V. harveyi* strain ATCC 14126^T^. It was routinely grown in artificial seawater (ASW) medium prepared by dissolving Instant Ocean^®^ sea salt (Instant Ocean Spectrum Brands, Blacksburg, VA, USA) in distilled water to obtain a final salinity of 35 g/L and supplemented with 0.1 M HEPES (Thermo Fischer Scientific Inc., Madrid, Spain) and 0.4% casein hydrolysate (Sigma-Aldrich, Madrid, Spain) to ensure the balanced growth of bacterial cultures. The pH of ASW medium was always adjusted to 7.0, 7.5, 8.0, 8.3 or 8.5 with NaOH or HCl, respectively, and then it was sterilized by filtration using TPP “rapid” 500 filtration unit (TPP Techno Plastic Products AG, Trasadingen, Switzerland). Analysis of *V. harveyi* growth and survival assays were performed in triplicate in 250 mL Erlenmeyer flasks beforehand cleaned with H_2_SO_4_ (97%, *v*/*v*), rinsed with deionized water and heated at 250 °C for 24 h to avoid any presence of residual organic substances.

### 2.2. Growth Curves

To prepare inocula, *V. harveyi* cells were grown in ASW supplemented with 0.4% casein hydrolysate and 0.1 M HEPES at pH 7.0, 7.5, 8.0, 8.3 or pH 8.5 overnight and 200 μL of the overnight culture was used to inoculate 20 mL fresh sterile ASW medium with the same composition and pH. The inoculated medium was further incubated at 20, 25 and 30 °C with shaking (90 rpm) and aliquots of cell cultures were periodically withdrawn under aseptic conditions to measure optical density (OD) at 600 nm. The experiments were done in triplicates and the resulting means of OD values were plotted against the time to generate growth curves.

### 2.3. Survival Assays

*V. harveyi* cells were grown in ASW supplemented with 0.4% casein hydrolysate and 0.1 M HEPES at pH 7.0, 7.5, 8.0, 8.3 or pH 8.5 until the cultures reach the early stationary phase (24 h) at 20, 25 or 30 °C. Aliquots (1 mL) of these cultures (i.e., cultures grown at particular pH and temperature) were further diluted with 39 mL of the corresponding sterile ASW that had the same pH and temperature but lacked casein hydrolysate to get a final cell density close to 10^8^ cells mL^−1^. The resulting cell suspensions were further incubated in darkness with shaking (90 rpm) at 20, 25 and 30 °C for 35 days. Experiments were carried out in triplicate and aliquots were periodically withdrawn to measure OD and to estimate cell size and culturability.

### 2.4. Enumeration of Cells and Estimation of Cell Size

Culturability, expressed as colony-forming units, was evaluated by spreading aliquots of *V. harveyi* suspensions withdrawn during the survival assays (and consecutively diluted with ASW) on Marine Agar (MA; PanReac AppliChem, Barcelona, Spain) followed by incubation for 24 h at 26 °C. To estimate the size of the cells, present in the control (overnight culture) and test samples and to determine the total number of cells in each sample, we used an epifluorescence microscope Nikon Eclipse E400 (Nikon Corporation, Tokio, Japan) equipped with a high-resolution video camera (Hamamatsu C2400, Hamamatsu Photonics, Hamamatsu City, Japan).

Both enumeration of cells and measurements of bacterial size were carried out via image analysis of cells fixed with 3.7% formaldehyde as described by Rosenberg et al. [26]. Briefly, aliquots of the fixed cells were stained with propidium iodide (0.001 mg/mL) (Thermo Fischer Scientific Inc., Madrid, Spain) and then filtered through 0.22 μm pore-size polycarbonate membrane filters (Merck Life Science S.L.U., Madrid, Spain). We used the standard protocol [27] that included filter staining with black dye prior to filtration. This step makes it possible to obtain more contrast images and eliminate autofluorescence. To determine the total number of cells, the individual cells were directly counted in each sample.

To estimate cell size, at least 20 areas in each filter with very flat fields containing enough bacteria and lacking very bright particles were selected to be digitized and analyzed by ImageJ 2.9.0 (National Institute of Health, Bethesda, MD, USA). For each sample, at least 200 bacteria were measured and, according to their length (μm), the cells were placed into four groups: ≤0.95; >0.95–≤1.3; >1.3–1.6≤; >1.6.

### 2.5. Statistical Analysis

All the experiments carried out in this study were performed in triplicate. The experimental data were processed to obtain the mean values and standard deviations indicated in each figure presenting the corresponding results. Statistical differences were assessed by using GraphPad Prism 8.0 software (GraphPad Software Inc., La Jolla, CA, USA).

Measured cell sizes were additionally processed using R package (version 4.2.2; R Core Team, 2022) to carry out statistical analysis and the graphical presentation of the results (GraphPad Prism 8.0; GraphPad Software). To statistically compare cell sizes at different incubation times, we constructed a series of Linear Mixed Models with a normal distribution of errors using the package “lmerTest” (version 3.1.3, [28]), one per pH and temperature tested. In these models, cell size was the dependent variable whereas time (as categorical term) was the explanatory one. In addition, we included replication as a random effect to control for the non-independence of the values obtained in each replicate experiment. In those models, in which day was statistically significant (*p* < 0.05), we compared the time-dependent differences between the different measurements using the package “emmeans” (version 1.8.5; [29]). The normality of residuals was determined graphically.

## 3. Results

### 3.1. Analysis of V. harveyi Growth at Different pH

**Figure 1:**
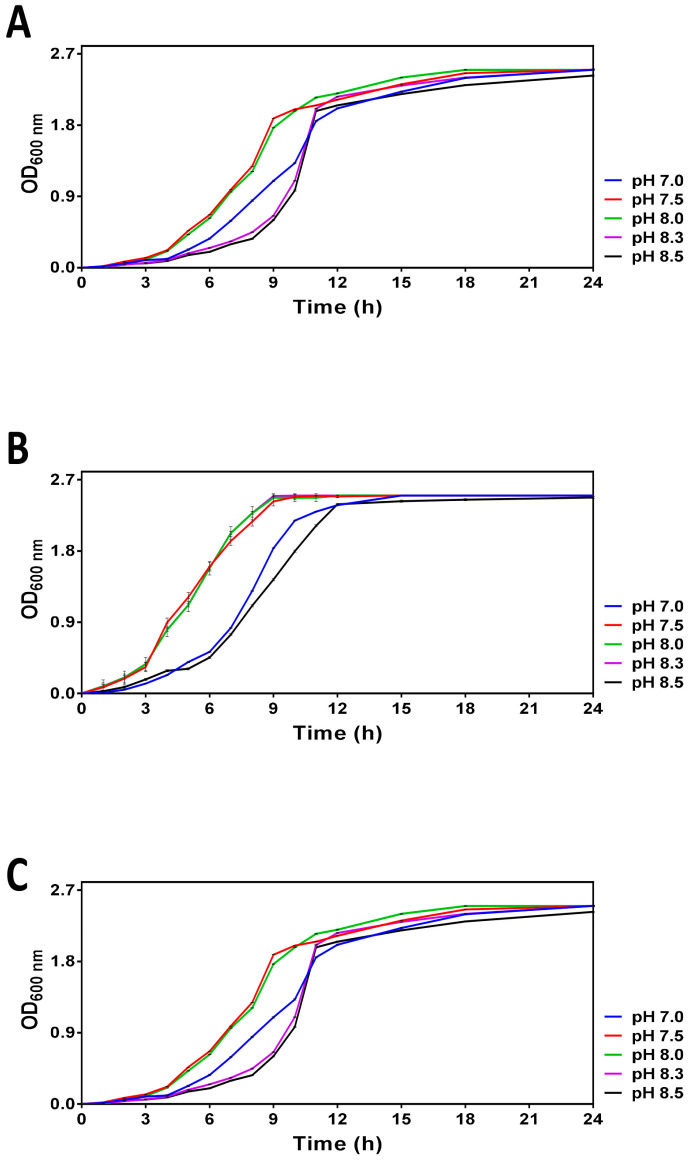
Growth curves obtained for *V. harveyi* grown in ASW medium with different pH (7.0, 7.5, 8.0, 8.3 and 8.5) at 20 °C (**Panel A**), 25 °C (**Panel B**) and 30 °C (**Panel C**) as described in Section 2. Optical density was regularly measured at 600 nm within 24 h. The mean values and standard deviation (indicated by vertical bars) were calculated based on the data obtained in 3 independent experiments.

The pH-dependent growth curves obtained for *V. harveyi* cultures incubated at 20, 25 and 30 °C are shown in Figure 1. These temperatures correspond to the summer average sea surface temperature in the Bay of Biscay (20 °C) in the Mediterranean Sea (25 °C) and in tropical areas (30 °C), respectively. The range of the tested pH was selected to mimic the pH of the world ocean in the pre-industrial era (pH 8.5), present time (ca. pH 8.0) and its upper limits predicted for 2100 and 2300 (i.e., 7.5 and 7.0, respectively). Although the overall growth profiles looked similar (i.e., the maximal optical density (>2.5) was reached in ca. 24 h following the entry into the stationary phase), we found that the pH of the media differentially affected the logarithmic growth. Moreover, the pH-dependent differences in *V. harveyi* growth are particularly well seen at 20 °C and 25 °C. Namely, the cultures at pH 7.5 and 8.0 enter the logarithmic phase of growth and reach the early stationary phase considerably faster (i.e., within the first 8 h) than those at other pHs, thus suggesting that more alkaline (8.3–8.5) and neutral (7.0) pH are less optimal for cell growth.

### 3.2. Assessing the Impact of pH on V. harveyi Adaptation under Limitation of Nutrients

The same temperatures (i.e., 20, 25 and 30 °C) and pH (i.e., 7.0, 7.5, 8.0, 8.3 and 8.5) conditions were used to analyze *V. harveyi* adaptation in ASW microcosms in the absence of carbon source. The changes underlying *V. harveyi* adaptation were assessed by monitoring optical density (see Section 3.2.1), enumerating the total number of bacteria and their culturable population (Section 3.2.2) and analyzing cell size and morphology (Section 3.2.3) as depicted in Supplementary Figure S1.

#### 3.2.1. Monitoring of Optical Density

**Figure 2:**
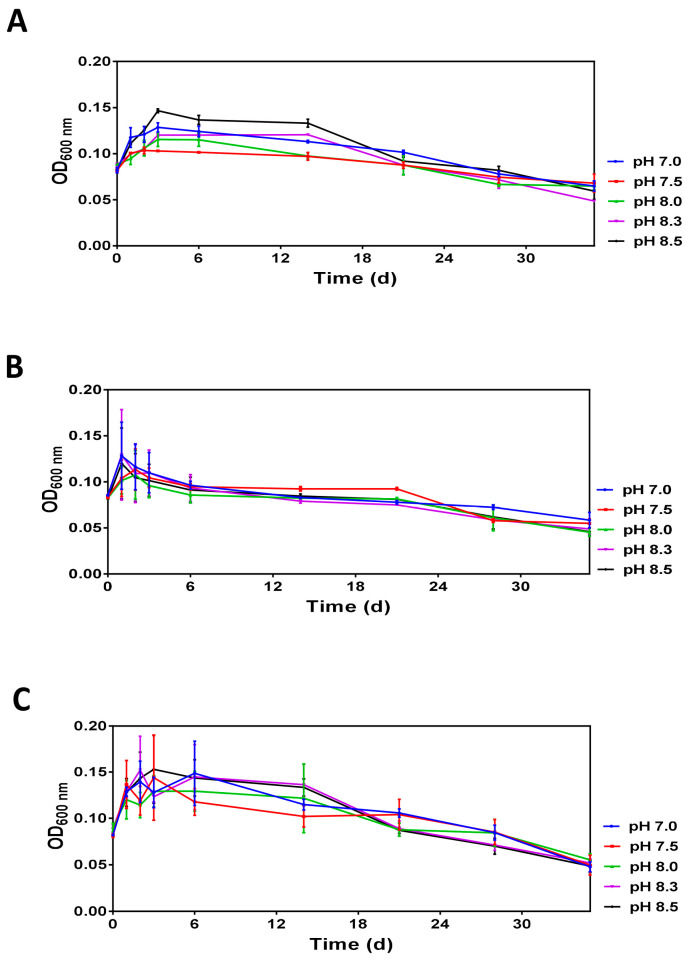
The time-dependent changes in the optical density (OD_600 nm_) of *V. harveyi* populations incubated at 20 °C (**panel A**), 25 °C (**panel B**) and 30 °C (**panel C**) Experiments were performed with samples obtained after dilution of overnight cultures with ASW supplemented with 0.1 M HEPES at pH 7.0, 7.5, 8.0, 8.3 and 8.5. The mean values and standard deviation (indicated by vertical bars) were calculated based on the data obtained in 3 independent experiments. The pH was measured at each time point, and it remained unchanged.

Analysis of optical densities over 35 days revealed similar profiles demonstrating that after a transient increase during the first three days, the optical density gradually decreases reaching the minimal values at day 35 (Figure 2). Moreover, the initial increase and subsequent decreases in optical densities are pH-dependent and the differences caused by pH were more prominent at 20 °C and 30 °C.

#### 3.2.2. Enumeration of Total Bacteria and Their Culturable Fraction

**Figure 3:**
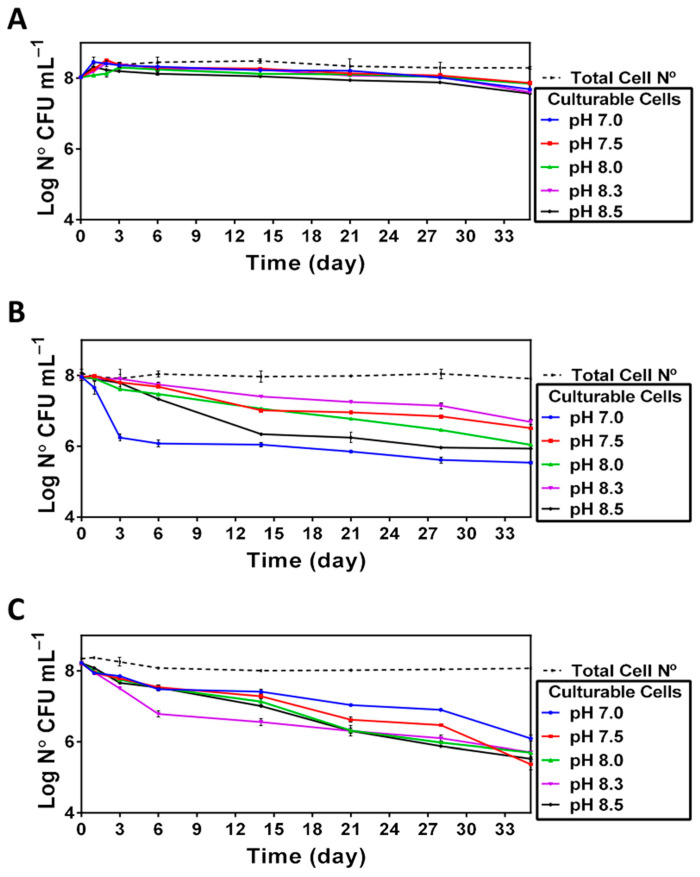
Culturability of *V. harveyi* (Log CFU mL^−1^) at 20 °C (**Panel A**), 25 °C (**Panel B**) and 30 °C (**Panel C**). Culturability and total bacterial count were determined as described in Section 2 for cultures incubated at different temperatures (20, 25 and 30 °C) and different pH (7, 7.5, 8, 8.3 and 8.5). The mean values and standard deviation (indicated by vertical bars) were calculated based on the data obtained in 3 independent experiments.

The results of enumeration (Figure 3) indicate that the total number of cells remained nearly unchanged for all the pHs tested in this study. In contrast, the number of culturable cells was readily affected in a pH- and temperature-dependent manner (Figure 3). Namely, although incubation of *V. harveyi* at 20 °C did not cause significant changes in the capacity of cells to resume growth after prolonged incubation, the use of higher temperatures (i.e., 25 °C and 30 °C) had a negative impact on cell culturability in a pH-dependent manner, indicating that the neutral pH was the least optimal one for preserving the cell capacity to grow.

#### 3.2.3. Analysis of Cell Size and Morphology

Although the total number of cells in each survival assay remained nearly the same, the gradual reduction in cell density suggests that the lower optical densities in cell cultures are likely linked to a reduction of cell size. Indeed, we found that *V. harveyi* incubation in ASW led to a reduction of cell size yielding an increasing fraction of cells with a coccoid-like morphology (for examples, see Supplementary Figure S2).

**Figure 4:**
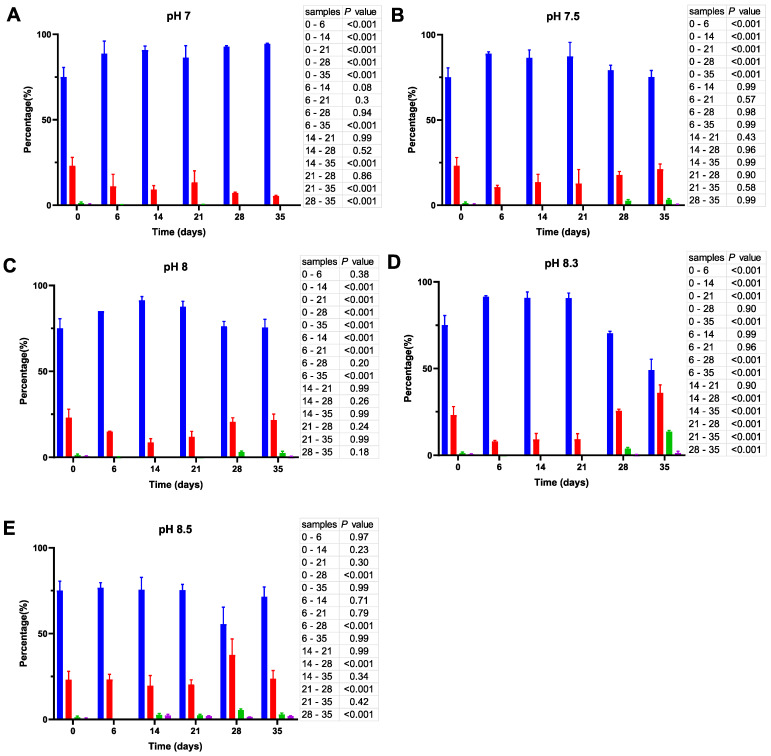
Cell size distribution at 20 °C. Cells are grouped according to their measured size ≤0.95 (█); >0.95–≤1.3 (█); >1.3–1.6≤ (█); >1.6 (█). The blue bars represent the population of coccoid-like cells (≤0.95 μm), whereas the cells with intermediate size and rod-like cells were arbitrarily placed in three additional groups indicated by red, green and violet bars. The data are mean values from three independent experiments with error bars representing the standard deviations. Statistical tests were performed to carry out the pairwise comparisons of the cell sizes datasets and the corresponding *p*-values are shown on the right of each panel.

**Figure 5:**
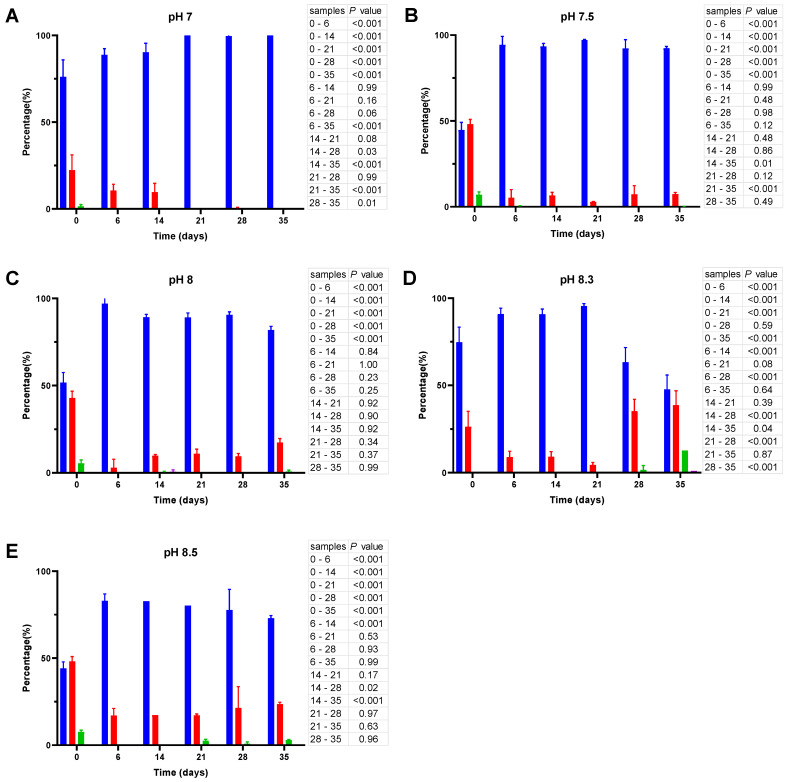
Cell size distribution at 25 °C. Cells are grouped according to their measured size ≤0.95 (█); >0.95–≤1.3 (█); >1.3–1.6≤ (█); >1.6 (█). The blue bars represent the population of coccoid-like cells (≤0.95 μm), whereas the cells with intermediate size and rod-like cells were arbitrarily placed in three additional groups indicated by red, green and violet bars. The data are mean values from three independent experiments with error bars representing the standard deviations. Statistical tests were performed to carry out the pairwise comparisons of the cell sizes datasets and the corresponding *p*-values are shown on the right of each panel.

**Figure 6:**
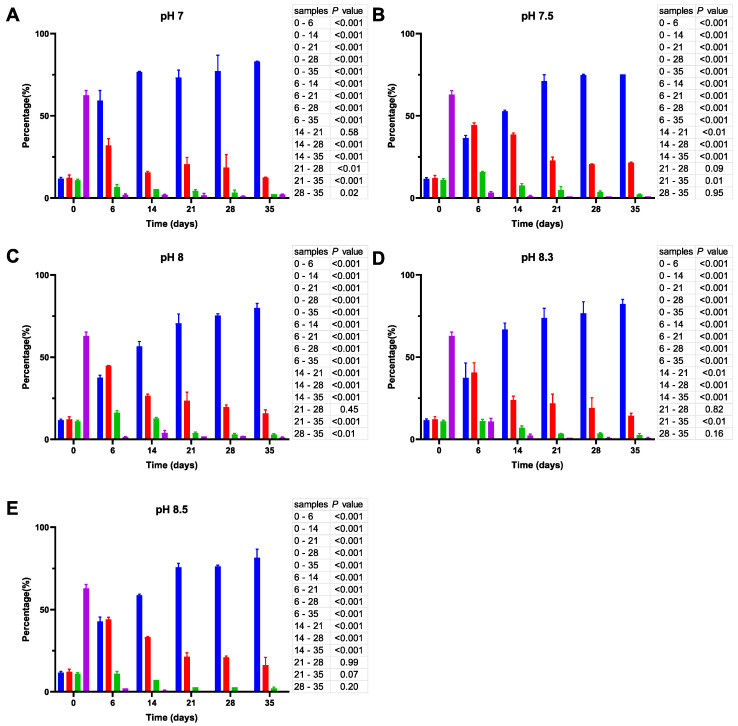
Cell size distribution at 30 °C. Cells are grouped according to their measured size ≤0.95 (█); >0.95–≤1.3 (█); >1.3–1.6≤ (█); >1.6 (█). The blue bars represent the population of coccoid-like cells (≤0.95 µm), whereas the cells with intermediate size and rod-like cells were arbitrarily placed in three additional groups indicated by red, green and violet bars. The data are mean values from three independent experiments with error bars representing the standard deviations. Statistical tests were performed to carry out the pairwise comparisons of the cell sizes datasets and the corresponding *p*-values are shown on the right of each panel.

Moreover, although many coccoid-like cells in the initial inoculate have a size of 0.77 ± 0.19 µm, prolonged incubation reduces the average size of these cells to 0.55 ± 0.034 µm. To present the dynamics of cell size changes in a quantitative manner, cell images were processed by using ImageJ and the resulting cell size data were further subjected to statistical analysis and presented in Figure 4, Figure 5 and Figure 6.

As shown in Figure 4, incubation of *V. harveyi* cells at 20 °C promotes the reduction of their size during the first two weeks and this effect is observed at nearly all pH values except at pH 8.5. In other words, the percentage of coccoid-like cells (≤0.95 µm) increases up to 85–90% after 6–14 days and this result is supported by statistical data (*p* < 0.001). However, incubation for a longer time (21–35 days) reverses this effect, thus leading to a subsequent increase in the number of larger cells (>0.95 μm), especially at more alkaline pH (i.e., at pH 8.3 and 8.5).

Unlike the initial inoculates used in ASW microcosms at 20 °C, the inoculates prepared at 25 °C have a considerably higher percentage (~50%) of bacillus-like cells (see cell size distribution at time 0; Figure 5). Similar to their counterparts at 20 °C, the cells undergo a gradual reduction in size, but it proceeds much faster at 25 °C, causing a significant decrease (*p* < 0.001) in the percentage of the bacillus-like bacteria after one week. Moreover, although the proportion of coccoid-like cells increases at pH 7 (Figure 5A), it reaches 100% after 21 days and remains unchanged thereafter, whereas the cell size reduction at more alkaline pH is partly reversible. As a result, we observed an increase in the percentage of larger cells by day 35. The effect is particularly prominent at pH 8.3 (Figure 5D).

Finally, we found that the size distribution of *V. harveyi* cells in the inoculate (day 0) grown at 30 °C (Figure 6) was different from those incubated at 20 and 25 °C. Namely, it contains considerably fewer coccoid-like cells, whereas the rest of the population is primarily represented by larger bacillus-like cells. Although incubation at this temperature likewise promotes cell size reduction, this process continues for five weeks at all pHs.

## 4. Discussion

A number of recent studies suggest that the current rate of CO_2_ emission will continue impacting the world ocean, thereby further lowering its pH to 7.0 by the end of 2300 (RCP 8.5). The ongoing increase of the sea surface temperature along with ocean acidification can cause many negative impacts, including the extinction of some species and the appearance of invasive ones. To assess the joint impact of environmental factors on *Vibrio* spp. in the context of climate change, we used *V. harveyi* as a model organism to study its adaptation at different pH and temperatures. The range of pH resembled the pH of the world ocean in the pre-industrial time (pH 8.3 and 8.5), its current value (pH 8.1), as well as the pH values predicted for 2100 and 2300 (i.e., 7.5 and 7.0, respectively). Moreover, the temperatures were selected to mimic the summer average sea surface temperature in the Bay of Biscay (20 °C), in the Mediterranean Sea (25 °C) and in tropical areas (30 °C).

The experiments assessing the combined effect of temperature and pH on *V. harveyi* growth in ASW medium revealed that both parameters differentially affect the growth curves (Figure 1). Namely, higher temperatures seem to promote *V. harveyi* growth manifested by its faster entry into the logarithmic (log) phase of growth in a pH-dependent manner under nutrient-rich conditions (see panel C *versus* panels A and B in Figure 1). Moreover, the faster entry into the log phase was observed at pH 7.5–8.0. Despite the capacity of 0.1 M HEPES to preserve pH constant during the logarithmic growth, it fails to maintain the initial pH once the cultures reach the stationary phase, thus resulting in pH drifting to 8.3, i.e., the average pH value recorded for the world ocean in the pre-industrial time [30]. This observation suggests that *V. harveyi* triggers some mechanisms able to alkalinize the surrounding medium. Indeed, the species that belong to the *Vibrio* genus possess multiple ways to cope with adverse pH. For example, Tanaka et al. (2008) discovered that *V. parahaemolyticus* can adapt to severe acid stress by accumulating products of lysine decarboxylation [31]. Although the drifting pH did not permit the preservation of the initial pH in the stationary phase of growth, our pilot experiments revealed that dilution of overnight cultures with ASW (1:40) made it possible to overcome the instability of this parameter. In other words, the experimental system obtained by dilution of overnight cultures with ASW preserved its original pH and, therefore, enabled the carrying out of survival assays with non-dividing *V. harveyi* cells in ASW microcosms.

Analysis of diluted (1:40) *V. harveyi* cultures revealed that *V. harveyi* survival at different temperatures and pH in ASW microcosms is characterized by a transient increase in optical density followed by its gradual decrease, potentially caused by a reduction of cell size (Figure 2). The initial increase of optical density is likely caused by dilution per se and can be associated with the altered concentration of environmental factors (e.g., oxygen) or biomolecules (e.g., autoinducers) probably affecting cell growth.

Although prolonged incubation under nutrient-limiting conditions leads to cell size reduction (see below) and accounted for the observed decrease of optical density (Figure 2), the total cell number remains nearly the same during the incubation period (Figure 3). In contrast, we found that *V. harveyi* culturability was differentially affected by the pH and temperature conditions used in the survival assays (Figure 3). While the culturability remains virtually constant over the entire incubation period at 20 °C (Figure 3A), it becomes greatly altered at higher temperatures (25 °C, Figure 3B and 30 °C, Figure 3C). Interestingly, although the culturability at higher temperatures is decreased in a pH-dependent manner, the effect of pH is different at 25 °C and 30 °C. Namely, it decreases at 25 °C as the pH approaches neutrality, resulting in a swift decline in the number of culturable cells at pH 7.0 (Figure 3B). Although the actual mechanisms accountable for this effect remains unknown, it seems likely that neutral pH might facilitate the fast loss of culturability due to accelerated acquisition of the VBNC state (see [21]) consequently, yielding an “equilibrium” population composed of both culturable and non-culturable cells. Consistent with the results obtained at 25 °C in our work, Yoon et al. [32] demonstrated that the fast loss of *Vibrio* culturability at neutral or acid pH can also occur at low temperatures close to 4 °C. On the contrary, the reduced culturability at 30 °C (Figure 3C) seems to be mainly caused by high temperature, and the pH in this case appears to play a minor role. This observation is reminiscent of our previous finding demonstrating a highly negative impact of high temperature (i.e., 30 °C) on *V. harveyi* survival in seawater microcosms [33]. It seems likely that an increase in populations of cells unable to resume growth after exposure to stress could be related to their ability to enter a state of dormancy, such as a VBNC state [21].

When exposed to some stress conditions bacteria can respond by shrinking their size, thereby reducing surface to volume ratio [34]. This adaptation mechanism has been documented in several studies [35,36,37]. Moreover, cell size reduction is accompanied by profound reprogramming of gene expression [22] downregulating numerous genes involved in central carbon metabolism, major biosynthetic pathways and energy production as well as upregulation of genes controlling recycling of macromolecules and acquisition of iron. Thus, the major role of these adaptation changes pursues the aim to reduce the need for carbon sources and minimize energy expenses. Here, we show that the same strategy (i.e., cell size reduction) is also employed by *V. harveyi* in the survival tests. Although the initial adaptation steps include a gradual reduction of cell size and accumulation of coccoid-like cells, the starting inoculates and dynamics of morphological changes are greatly affected by both temperature and pH. In particular, we found that the inoculates prepared by growing *V. harveyi* at higher temperatures have a considerably higher (especially at 30 °C) percentage of larger cells including rod-like ones (Figure 6 versus Figure 4 and Figure 5), thus suggesting that higher incubation temperature leads to larger cells in nutrient-rich environments. However, once the nutrients become scarce (i.e., upon dilution of cell cultures with fresh ASW lacking carbon sources), bacteria undergo fast morphological changes and acquire a coccoid-like phenotype (Figure 4, Figure 5 and Figure 6). Moreover, the percentage of coccoid-like cells becomes particularly high at low pH and 25 °C (Figure 5A). Another interesting observation concerns the appearance of larger cells, especially at 20 °C and 25 °C after 28 days of incubation. The effect is particularly prominent at pH resembling its pre-industrial values, i.e., at pH 8.3–8.5 (panels D and E). It seems likely that vibrios at pH 8.3 and pH 8.5 (“pre-industrial” pH) are less stressed than those at lower pH and, therefore, the entire population is more dynamic. Therefore, the observed increase in cell size at the end of the incubation period could be a result of the “bust and boom” mechanism [21], which implies the death of a small fraction of the bacterial population to provide nutrients to the remaining cells.

Taken together, our findings demonstrate that *V. harveyi* can differentially tolerate a wide range of pH corresponding to past, current and future pH of the world ocean. According to our results, in ecosystems where the average sea temperature is around 20 °C, seawater acidification on its own should not be a serious threat, at least for *V. harveyi*. However, since ocean acidification is accompanied by increases in the average sea surface temperature, the impact of lower pH can be more significant, especially at 25 °C. Our finding that lower pH at this temperature, in particular, pH 7.0, has a strong negative impact on *V. harveyi* culturability and potentially facilitates the acquisition of VBNC state, can be explored to reproduce similar conditions in shrimp or bivalves aquaculture to considerably reduce the presence of active *Vibrio* pathogens and, therefore, decrease the risk of production of contaminated seafood and human infections. According to RCP 8.5 predictions [3], sea surface temperature is estimated to increase by 2.73 ± 0.73 °C by the end of this century, thus suggesting that even the Mediterranean Sea temperature can occasionally rise close to 30 °C and the pH drop to 7.5. Nevertheless, our results show that pH plays a less important role than temperature, because, when the temperature approaches 30 °C, it will likely act as a primary stress factor determining cell adaptation and survival. In addition, our results suggest that, despite the apparently crucial role of the coccoid-like phenotype in conferring cell resistance to suboptimal pH and temperatures, *V. harveyi* (and likely other species) can potentially rely on other adaptation strategies likely involving the acquisition of the VBNC state or exploring the “boost and boom” mechanisms.

Finally, although this study was primarily focused on the pH-dependent adaptation at different temperatures, the tolerance of *V. harveyi* to different pH is arguably dependent on other factors (e.g., salinity, visible light and/or predators) not only influencing its adaptation and fitness in seawater microcosms [36,38] but also affecting *Vibrio* dynamics in natural aquatic systems [39], and, therefore, the individual contribution of these factors to the pH-dependent adaptation of *Vibrio* spp. would be important to address in future studies.

## Data Availability

All data are provided in full in this paper.

## Supplementary Materials

The following supporting information can be downloaded at: https://www.mdpi.com/article/10.3390/microorganisms11041075/s1, Figure S1: Analysis of *V. harveyi* cells from survival assays; Figure S2: Examples of *V. harveyi* cells that possess different morphology at pH 8.5 and 30 °C. The images correspond to the cells present in the control sample (panel A) and population obtained after incubation for 21 days (panel B). Several cells of different sizes indicated by arrows: ≤ 0.95 (I); > 0.95–≤ 1.3 (II); >1.3–1.6 ≤ (III); >1.6 (IV).
