# Supplementary material for: Addressing the Joint Impact of Temperature and pH on Vibrio harveyi Adaptation in the Time of Climate Change
Source: Microorganisms. 2023 Apr 20;11(4):1075. doi: 10.3390/microorganisms11041075 (PMC10142252; doi:10.3390/microorganisms11041075)
Supplement: Supplementary file 1 [file microorganisms-11-01075-s001.zip › microorganisms-2165357-supplementary.pdf]

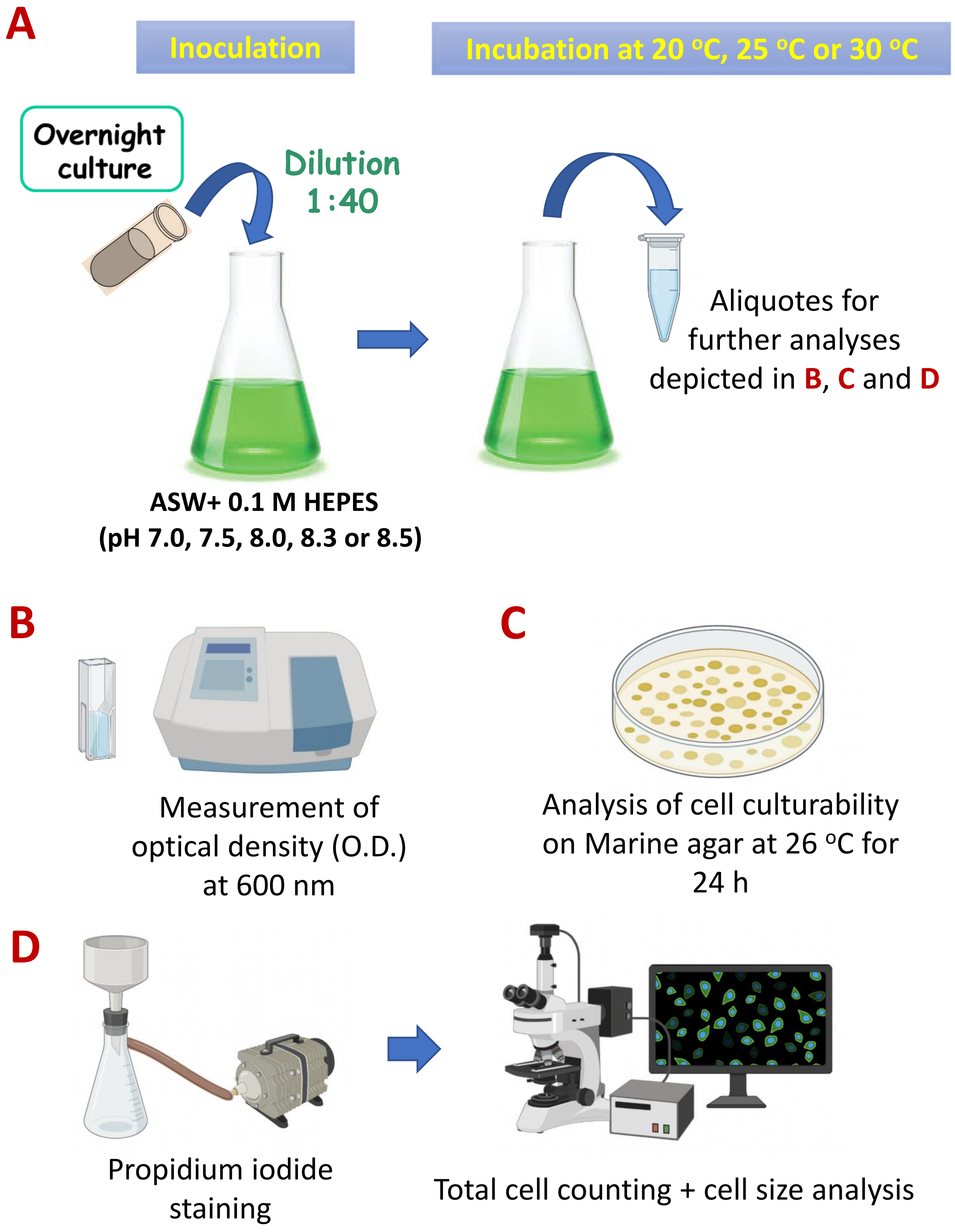

Figure S1. Analysis of *V. harveyi* cells from survival assays

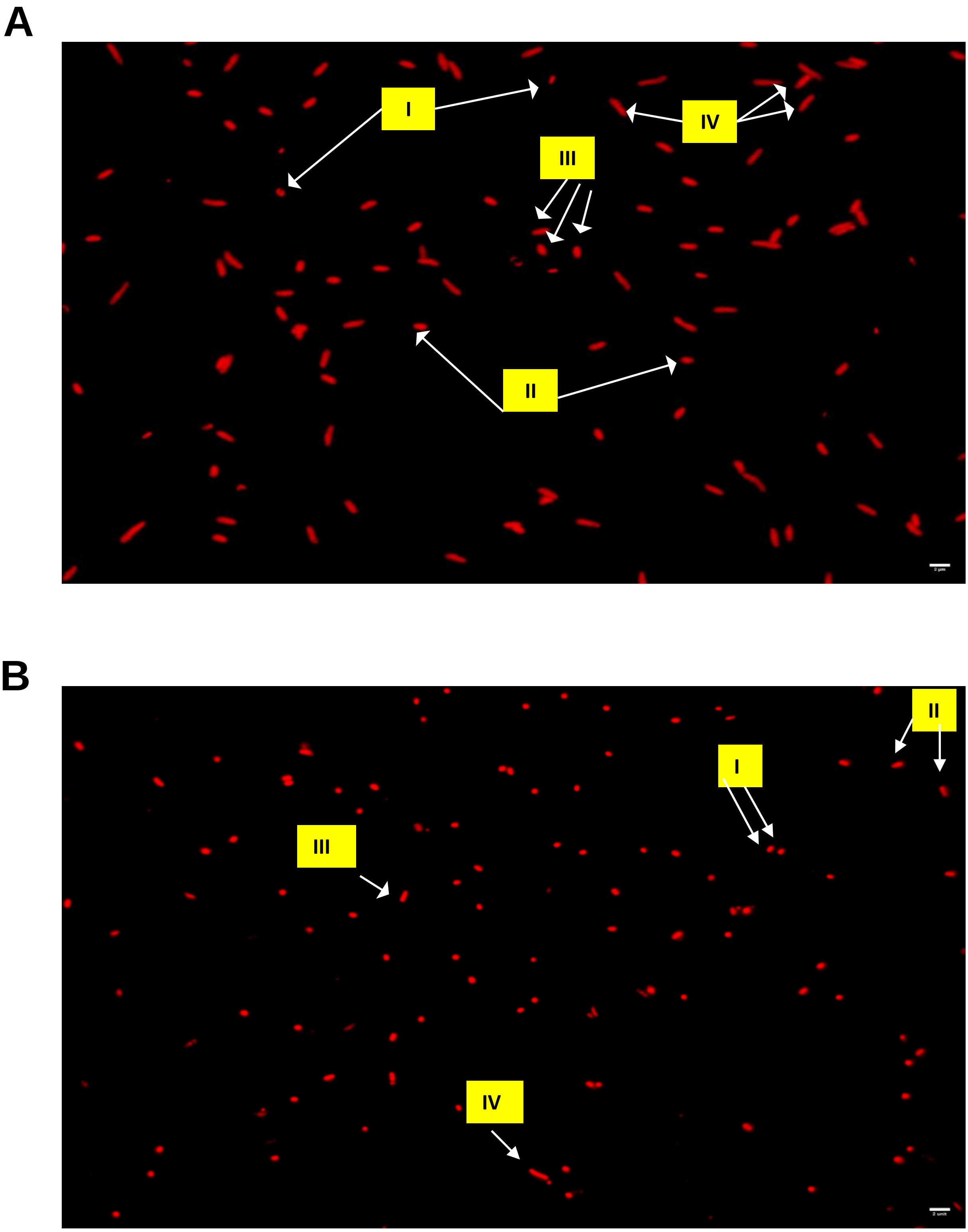

Figure S2. Examples of *V. harveyi* cells that possess different morphology at pH 8.5 and 30 ^{o}C. The images correspond to the cells present in the control sample (panel A) and population obtained after incubation for 21 day (panel B). Several cells of different sizes indicated by arrows: ≤ 0.95 (**I**); > 0.95 - ≤ 1.3 (**II**); >1.3 -1.6 ≤ (**III**); >1.6 (**IV**).
